# Quantifying Asymmetry in Gait: The Weighted Universal Symmetry Index to Evaluate 3D Ground Reaction Forces

**DOI:** 10.3389/fbioe.2020.579511

**Published:** 2020-10-23

**Authors:** Sónia A. Alves, Rainald M. Ehrig, Peter C. Raffalt, Alwina Bender, Georg N. Duda, Alison N. Agres

**Affiliations:** ^1^Julius Wolff Institute, Charité –Universitätsmedizin Berlin, Corporate Member of Freie Universität Berlin, Humboldt-Universität zu Berlin, and Berlin Institute of Health, Berlin, Germany; ^2^Zuse Institute Berlin, Berlin, Germany; ^3^Department of Physical Performance, Norwegian School of Sport Sciences, Oslo, Norway

**Keywords:** gait asymmetry, symmetry, ground reaction forces, bilateral signals, crutch gait

## Abstract

Though gait asymmetry is used as a metric of functional recovery in clinical rehabilitation, there is no consensus on an ideal method for its evaluation. Various methods have been proposed to analyze single bilateral signals but are limited in scope, as they can often use only positive signals or discrete values extracted from time-scale data as input. By defining five symmetry axioms, a framework for benchmarking existing methods was established and a new method was described here for the first time: the weighted universal symmetry index (*wUSI*), which overcomes limitations of other methods. Both existing methods and the *wUSI* were mathematically compared to each other and in respect to their ability to fulfill the proposed symmetry axioms. Eligible methods that fulfilled these axioms were then applied using both discrete and continuous approaches to ground reaction force (GRF) data collected from healthy gait, both with and without artificially induced asymmetry using a single instrumented elbow crutch. The *wUSI* with a continuous approach was the only symmetry method capable of identifying GRF asymmetry differences in different walking conditions in all three planes of motion. When used with a continuous approach, the *wUSI* method was able to detect asymmetries while avoiding artificial inflation, a common problem reported in other methods. In conclusion, the *wUSI* is proposed as a universal method to quantify three-dimensional GRF asymmetries, which may also be expanded to other biomechanical signals.

## Introduction

Unilateral lower limb injuries and degenerative musculoskeletal diseases often lead to asymmetries in applied loads during movement (McCrory et al., 2001; Shakoor et al., 2011; Queen et al., 2015). Across many disciplines, gait asymmetry is used as a metric to evaluate pathology status, as longitudinal measurements give insight into patients' functional recovery or disease progression (Aqil et al., 2016; Wesseling et al., 2018). Such assessments of gait asymmetry can also be used to evaluate and compare different interventions that target the underlying disease (Bohm et al., 2016) and are frequently linked to patients' clinical outcomes (Farkas et al., 2019). The progression to full limb loading from unilateral limb unloading is an indicator for musculoskeletal or neuromuscular regeneration and recovery (Duda et al., 2002), with direct effects on tissues (Birkhold et al., 2017; Albiol et al., 2020). Reduced mechanical loading can lead to bone loss (Jørgensen et al., 2000), whereas increased loading may enhance the risk of developing degenerative musculoskeletal pathologies (Block and Shakoor, 2010; Jones et al., 2013; Gustafson et al., 2019). As a result, restoration of gait asymmetry is not only an indicator of functional recovery but also an important aim for clinical rehabilitation practice. Yet to date, rehabilitation programs lack precise guidance, as there is no consensus on how to effectively quantify such asymmetries (Lauziere et al., 2014; Viteckova et al., 2018).

Gait asymmetry metrics of both healthy (Herzog et al., 1989) and injured (McCrory et al., 2001; Queen et al., 2015; Wiik et al., 2017) individuals can rely on assessments of kinematics (e.g., joint angles) as well as kinetics (e.g., ground reaction forces, or GRF). Due to the ease of data collection, GRF have been commonly used to assess gait asymmetry in unilaterally injured patients (McCrory et al., 2001; Aqil et al., 2016; Wiik et al., 2017). A number of available methods such as the Symmetry Index (*SI*) (Robinson et al., 1987), Ratio Index (*RI*) (Ganguli et al., 1974), the Symmetry Angle (*SA*) (Zifchock et al., 2008) or the recently introduced Normalized Symmetry Index (Queen et al., 2020) use collected GRF data to determine gait asymmetry. Yet major technical limitations in these methods have been described, such as artificial inflation (Herzog et al., 1989; Błazkiewicz et al., 2014), which excludes comparisons of irregular signals that are common in pathological gait. Thus, the utility of existing methods to assess gait asymmetry across various severities of injury and disease is particularly limited in scope.

A further limitation of the existing methods is the use of discrete approaches to compare bilateral signals (Viteckova et al., 2018), where singular values such as peak magnitudes (Burnett et al., 2011) or integrals (Wiik et al., 2017) are usually extracted from GRF data. However, a single discrete metric derived from the GRF may oversimplify the dynamics of human patient gait patterns (Schöllhorn et al., 2002; Sole et al., 2017). Such discrete approaches often discard relevant, time-scale information that may be needed to identify pathological characteristics of movement patterns and may affect the entire gait cycle (Schöllhorn et al., 2002; Sole et al., 2017). Alternative approaches exist, such as those that include the entire time series (here defined as a “continuous” approach), but so far have been only minimally explored (Viteckova et al., 2018). These continuous approaches are more robust, with a lower probability of false positives than discrete analyses of the same data (Pataky et al., 2016). It remains unclear if existing methods that traditionally calculate asymmetry from GRF data with a discrete approach could be further applied using a continuous approach.

The mediolateral and anteroposterior components of the GRF are often neglected in gait asymmetry analyses. Most methods, even when continuously applied, only analyze the vertical component of the GRF. This is likely for simplification of the analysis, due to mathematical irregularities that occur when asymmetric data exhibit values that can be both positive and negative in the same waveform. However, the exclusion of these two additional GRF components removes important insights into pathological gait, as these planes of motion are critical in dynamic balance strategies (Richards et al., 2013) often found in such cohorts. Though such tools could greatly benefit clinical gait analyses, a method to assess GRF asymmetry with a continuous approach in the mediolateral and anterior axes remains unestablished.

The primary aim of this work was to determine if a single method could universally analyze and identify gait asymmetry across all three-dimensional GRF signals. A further aim was to define specific benchmarking standards for an appropriate and effective symmetry method. In order to address these aims, a three-step analysis was performed with existing methods, along with a new method established within this study. In the first step, five symmetry axioms were proposed, all of which must be satisfied for a symmetry method to be considered for further analysis. Second, through mathematical analyses, we evaluated available symmetry methods based on the proposed symmetry axioms. Hereby we presented a novel method—the weighted universal symmetry index (*wUSI*)—that allows to quantify gait asymmetry in a manner that both overcomes limitations identified in previous methods and fulfills the above defined symmetry axioms. Third, to test the utility of the symmetry method, only those methods that fulfilled the proposed axioms were then applied to three-dimensional GRF data gathered from healthy subjects, using both a discrete and continuous approach. In this experimental setup, the GRF asymmetry was controlled and artificially induced by means of unilateral crutch-assisted walking, allowing to switch asymmetry “on” and “off.” We hypothesized that with use of an appropriate method, the crutch-assisted walking condition would exhibit higher asymmetry in all three components of the GRF.

## Methods

### Symmetry Axioms

If any method included in this study fulfilled the following five axioms, it was then considered here as an appropriate symmetry method and included for further analyses. The method must (1) quantify asymmetry magnitude in a finite range, i.e., a lower and upper bound should exist to consider a maximum asymmetry (a finite range of [−1, 1] was selected here for mathematical analyses); (2) identify perfect symmetry and complete asymmetry, which indicates the values for both limbs where a scenario of perfect symmetry (*S* = 0) and complete asymmetry (*S* = −1 *or* 1) may exist; (3) identify the direction of asymmetry, which allows for identification of the most loaded side; (4) exhibit signal selection independency, as the absolute value of the symmetry measure should be independent of the order of selection (e.g., left over right limb in healthy subjects), thus not affecting the final symmetry measure; and (5) exhibit scaling invariance, as changes in the scale should not yield changes in the symmetry measure.

In the following, we consider *x* and *y* as input variables for the symmetry methods, where each variable corresponds to either the left or right side. The input variable, for instance, can be any bipedal kinetic or kinematic parameter (e.g., mediolateral ground reaction forces). If both signals *x* and *y* are always positive, we can summarize these axioms in Equations (1–5):

(1) $$\begin{array}{l} \text{Finite range}:\;S\left(x,y\right)\in \left[-1,1\right] \end{array}$$

(2) $$\begin{array}{l} \text{Perfect symmetry and asymmetry}:\;S\left(x,x\right)=0,\;S\left(x,0\right)=1,\;\\ \;S\left(0,y\right)=-1 \end{array}$$

(3) $$\begin{array}{l} \text{Symmetry direction identification}:\;x\geq y\Rightarrow S\left(x,y\right)\geq 0;\\ \;x\leq y\Rightarrow S\left(x,y\right)\leq 0 \end{array}$$

(4) $$\begin{array}{l} \text{Signals order independency}:\;S\left(x,y\right)=-S\left(y,x\right) \end{array}$$

(5) $$\begin{array}{l} \text{Scaling invariance}:\;S\left(kx,ky\right)=S\left(x,y\right)\\ \text{ for an arbitrary k} \end{array}$$

If one or both signals contain also negative values or cross zero, axiom (Equation 2) is modified to (Equation 2') to determine perfect symmetry and complete asymmetry:

(2’) $$\begin{array}{l} \text{Perfect symmetry and asymmetry}:\;S\left(x,x\right)=0,\;S\left(x,-x\right)=1\\ \;for\;x\geq 0\; \end{array}$$

Thus, complete asymmetry now is given if *y* = −*x*.

Before analyzing different symmetry methods based on the defined axioms, an important consequence of the scaling invariance (Equation 5) should be noted. Let *k* = 1/*x*, then it immediately follows that *S*(*x, y*) = *S*(1, *y*/*x*) holds. Thus, any symmetry measure depends only on the *y* to *x* ratio. Defining φ by tan φ = *y*/*x*, the symmetry measure, *S*, can be expressed in terms of the angle φ. *S* may also be defined as *S*(φ) = *S*(1, arctan φ). To allow for direct comparison of the different methods selected, the five axioms may also be expressed in terms of the function *S*(φ).

If both *x* and *y* signals are positive, the angle φ is limited to [0, π/2]. The axioms (Equations 1–4) above are then modified as in Equations (6–9):

(6) $$\begin{array}{l} S\left(\varphi \right)\in \left[-1,\;1\right] \end{array}$$

(7) $$\begin{array}{l} S\left(\pi /4\right)=0,\;S\left(0\right)=1,\;S\left(\pi /2\right)=-1 \end{array}$$

(8) $$\begin{array}{l} 0\leq \varphi \leq \pi /4\Rightarrow S\left(\varphi \right)\geq 0\text{ and }\pi /4\leq \varphi \leq \pi /2\Rightarrow S\left(\varphi \right)\leq 0 \end{array}$$

(9) $$\begin{array}{l} S\left(\varphi \right)=-S\left(\pi /2-\varphi \right) \end{array}$$

If *x* and *y* signals are positive and negative, the angle φ is limited to [0, 2π] and the conditions are more detailed as shown in Equations (10–13):

(10) $$\begin{array}{l} S\left(\varphi \right)\in \left[-1,\;1\right] \end{array}$$

(11) $$\begin{array}{l} S\left(\pi /4\right)=0,\;S\left(3\pi /4\right)=-1,\;S\left(5\pi /4\right)=0,\;\\ \;S\left(7\pi /4\right)=1 \end{array}$$

(12) $$\begin{array}{l} 0\leq \varphi \leq \frac{\pi }{4}\Rightarrow S\left(\varphi \right)\geq 0,\;\frac{\pi }{4}\leq \varphi \leq \frac{5\pi }{4}\Rightarrow S\left(\varphi \right)\leq 0\\ \text{ and }\frac{5\pi }{4}\leq \varphi \leq 2\pi \Rightarrow S\left(\varphi \right)\geq 0 \end{array}$$

(13) $$\begin{array}{l} S\left(\varphi \right)=-S\left(\varphi +\pi \right) \end{array}$$

**Figure 1:**
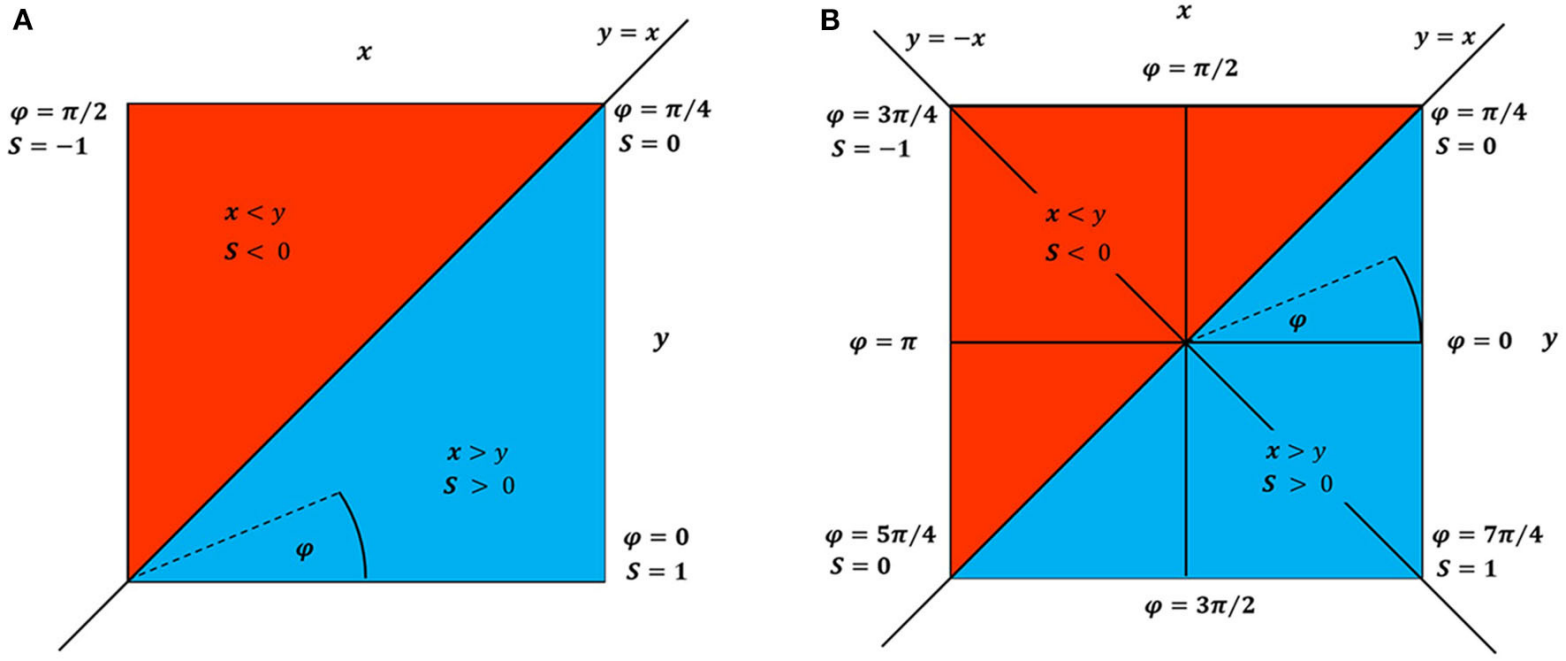
Schematic visualization for any symmetry measure (*S*) outcome according to the combination of only positive input values for *x* and *y* **(A)** and to combination of positive and negative *x* and *y* values **(B)**. The φ variable represents φ = arctan2(*y*/*x*). The blue shaded color represents the area of positive asymmetry values, i.e., *x* > *y* → *S* > 0 and the red shaded color represents the area of negative asymmetry values, i.e., *x* < *y* → *S* < 0. The diagonal common to both **(A)** and **(B)** (top-right toward bottom-left) and anti-diagonal in **(B)** (top-left toward bottom-right) represent the cases for *y* = *x* and *y* = −*x*, respectively.

To aid understanding of the axioms through visualization, Figure 1 represents the *S* measures for the different possible values of *x* and *y*, expressed by the angle φ for the case when both signals only contain positive values (Figure 1A) and when contain positive and negative values (Figure 1B).

### Symmetry Methods

Three commonly used methods to estimate asymmetry were included in the present study: *SI* (Robinson et al., 1987), *RI* (Ganguli et al., 1974) and *SA* (Zifchock et al., 2008), represented by Equations (14–16), respectively.

(14) $$\begin{array}{l} SI\left(x,y\right)=\frac{x-y}{x+y} \end{array}$$

(15) $$\begin{array}{l} RI\left(x,y\right)=1-\frac{x}{y} \end{array}$$

(16) $$\begin{array}{l} SA\left(x,y\right)\;=\;1-\frac{4}{\pi }\arctan\left(\frac{y}{x}\right) \end{array}$$

To facilitate comparisons, the methods were rescaled to satisfy the symmetry axiom (Equation 1). The *RI* method cannot be scaled to [−1, 1], since for fixed *y* values, *x* → ∞ and for fixed *x* values and *y* → 0, *RI* → −∞. Due to this, the *RI* does not fulfill the axioms and was no longer considered for analyses. However, the *SI* and *SA* methods can be rescaled and were expressed as a function of φ, allowing direct comparisons according to the axioms defined. The *SI* and *SA* are represented for positive signals in Equations (17, 18), respectively.

(17) $$\begin{array}{l} SI\left(\varphi \right)=\frac{\cos\varphi -\sin\varphi }{\cos\varphi +\sin\varphi } \end{array}$$

(18) $$\begin{array}{l} SA\left(\varphi \right)=1-\frac{4}{\pi }\varphi \end{array}$$

An essential modification of *SA* leads to a new method (Equations 19, 20) in terms of the angle φ and in terms of *x* and *y*, respectively. This method was defined here as the universal symmetry index (*USI*), and satisfied all the required axioms for positive signals.

(19) $$\begin{array}{l} USI\left(\varphi \right)=\cos\varphi -\sin\varphi \end{array}$$

(20) $$\begin{array}{l} USI\left(x,y\right)=\frac{x-y}{\sqrt{x^{2}+y^{2}}} \end{array}$$

If negative signal values also occur within the signal, i.e., the signals cross zero, the *SI* method cannot be further considered, since *SI* is undefined if *y* = −*x*. Thus, the *SI* was not further considered in the analyses, as it cannot be considered a universal method for all types of signals. Only two methods remained: *SA* and *USI*. Both methods must be rescaled to satisfy the axioms in Equations (10–13). This is presented in Equations (21, 22), which additionally yielded a case distinction for the *SA* method.

(21) $$\begin{array}{l} SA\left(\varphi \right)=\{\begin{array}{c} \frac{1}{2}-\frac{2\varphi }{\pi },\;\varphi \in \left[0,3\pi /4\right]\\ -1+\frac{2\varphi }{\pi },\;\varphi \in \left[3\pi /4,7\pi /4\right]\;\\ 1-\frac{2\varphi }{\pi },\;\varphi \in \left[7\pi /4,2\pi \right] \end{array} \end{array}$$

(22) $$\begin{array}{l} USI\left(\varphi \right)=\frac{\cos\varphi -\sin\varphi }{\sqrt{2}} \end{array}$$

Both methods can also be expressed in terms of *x, y*. For *SA*, it can be obtained by replacing φ in Equation (21) through *arctan*2(*y*/*x*). The *USI* method, in terms of *x, y*, is suitable for all type of signals is represented in Equation (23):

(23) $$\begin{array}{l} USI\left(x,y\right)=\frac{x-y}{\sqrt{2\left(x^{2}+y^{2}\right)}} \end{array}$$

All methods described here are sensitive to artificial inflation. This occurs if both signals are very small compared to the magnitude of the measurement errors, since all symmetry measures depend on the ratio of *x* to *y*. To overcome artificial inflation, a methodology was developed for the *USI* method, which is based on the construction of a weighting function *W*(*x, y*, σ) using a profile similar as a Cauchy distribution, represented in Equation (24). Multiplication of *USI* with this factor *W* yields the new weighted *USI (wUSI)* method, represented in Equation (25). For the variable σ, the minimum standard deviation value of the experimental data should be selected, otherwise real features of the symmetry results could be filtered out. Further details on the establishment of the *wUSI* method can be found in the Supplementary Material.

(24) $$\begin{array}{l} W\left(x,y,\sigma \right)=1\;-\;\frac{\sqrt{2}\;\sigma }{\sqrt{2\sigma^{2}+x^{2}+y^{2}}} \end{array}$$

(25) $$\begin{array}{l} wUSI\left(x,y,\sigma \right)\;=\;USI\left(x,y\right){\;}^{*}W\left(x,y,\sigma \right)\\ \;=\;USI{\left(x,y\right)}^{*}\left(1\;-\frac{\sqrt{2}\;\sigma }{\sqrt{2\sigma^{2}+x^{2}+y^{2}}}\right) \end{array}$$

In summary, based on the proposed symmetry axioms, only the *SA* and the newly proposed *wUSI* methods were considered further as eligible methods. These two methods were then applied to GRF signals from physiological gait data.

### Physiological Data

Fifteen healthy participants (seven females, eight males, age: 31.1 ± 5 years old, BMI: 23.4 ± 2.3 kg/m^2^) participated in this study. The local ethics committee approved the study and all subjects provided written informed consent (Ethikkomission der Charité, No. EA1/079/17). All investigations were performed in accordance with the Declaration of Helsinki. Two walking conditions were tested: unassisted walking and unilaterally assisted walking, which aimed to reduce the GRF during the stance phase of the gait cycle by offloading to a single instrumented elbow crutch.

Participants walked at a self-selected speed with their own sport shoes along a defined 10-m path. The trials were then repeated with the participants using the single instrumented elbow crutch on the dominant hand (right hand for all participants), adjusted to each individual participant's height by a physiotherapist. As the participants do not usually use walking aids, they were given time before data collection to practice proper use. The participants were instructed to walk with 2-point gait with a single crutch: the hand that holds the crutch followed the movement of the limb on the opposite side. The three components of the GRF (Fx: mediolateral, Fy: anteroposterior, and Fz: vertical) were collected with a sampling frequency of 1,000 Hz using two embedded force plates (AMTI-BP400600, Watertown, MA, USA). For each walking condition, each limb cleanly contacted the force plate six times, with the crutch avoiding contact with the force plate. The instrumentation system within the crutch has been described elsewhere (Damm et al., 2013). Briefly, load transducers (KM30z-2kN, ME-Meßsystem GmbH, Germany) were embedded into the crutch and measured applied forces along the crutch shaft. The load transducers were directly connected through the AD-card within a VICON Giganet Box (VICON, Oxford, UK) to allow for synchronous collection of GRF and crutch forces.

After data collection, each step was identified using a custom script in RStudio (Version 1.0.136, R. RStudio, Inc., Boston, MA, USA). Initial contact and toe off were identified when the vertical GRF component was above and below 10% of the subject's bodyweight (Abe et al., 2010), respectively. For each component, the mean curve of the GRF stance phase was computed across the six steps for the right and left lower limb using the dynamic time warping procedure previously described by Bender and Bergmann (2012). The symmetry outcomes for each walking condition were calculated using methods that fulfilled all symmetry axioms: the *SA* and the *wUSI*. Here, a [−100, 100] range was selected for the symmetry measures, allowing for interpretation of the *S* value of 1 as 100% of asymmetry. For each individual, the maximum values captured by the single instrumented elbow crutch were averaged across the six steps.

Using a custom script in MATLAB (R2019b, The Mathworks Inc., Natick, USA), both discrete and continuous approaches were applied to calculate the symmetry outcome. For the discrete approach, the impulse value of each stance phase was used for analysis in order to minimize any bias introduced by the variable selected, which includes information from the complete time series (Kean et al., 2012). Statistical analyses were conducted with IBM SPSS Statistics for Windows, version 25 (IBM Corp., Armonk, NY, USA) and Cohen's *d* estimated effect sizes were also determined (Cohen, 1988). For the continuous approach, the normalized stance phase was considered as a complete time series for each limb. For these continuous data sets, statistical parametric mapping (SPM) analyses were applied as described elsewhere (Friston et al., 1994; Pataky et al., 2013). Briefly, SPM uses random field theory to perform topological inference instead of performing separate inferential tests at each time point. A *p*-value is calculated for clusters of statistics that cross a critical threshold. If the threshold is crossed, the cluster has a *p* < 0.05, rejecting thus the null hypothesis (Friston et al., 1994; Pataky et al., 2013). The open-source spm1d package (www.spm1d.org, version M.0.4.7 of 27th November 2019) was used to perform the SPM analyses in Matlab as described by Pataky (2012). Prior to any inferential procedure, data normality was assessed with the Shapiro-Wilk test for the discrete approach and with the built-in function “spm1d.stats.normality.*t*-test” for the continuous approach. For both discrete and continuous approaches, a two-tailed, paired *t*-test was used to compare the symmetry outcomes walking conditions when the input data were normally distributed. In the case that the input data were not normally distributed, a non-parametric, paired *t*-test was performed.

## Results

### Mathematical Analyses of the Symmetry Methods

**Figure 2:**
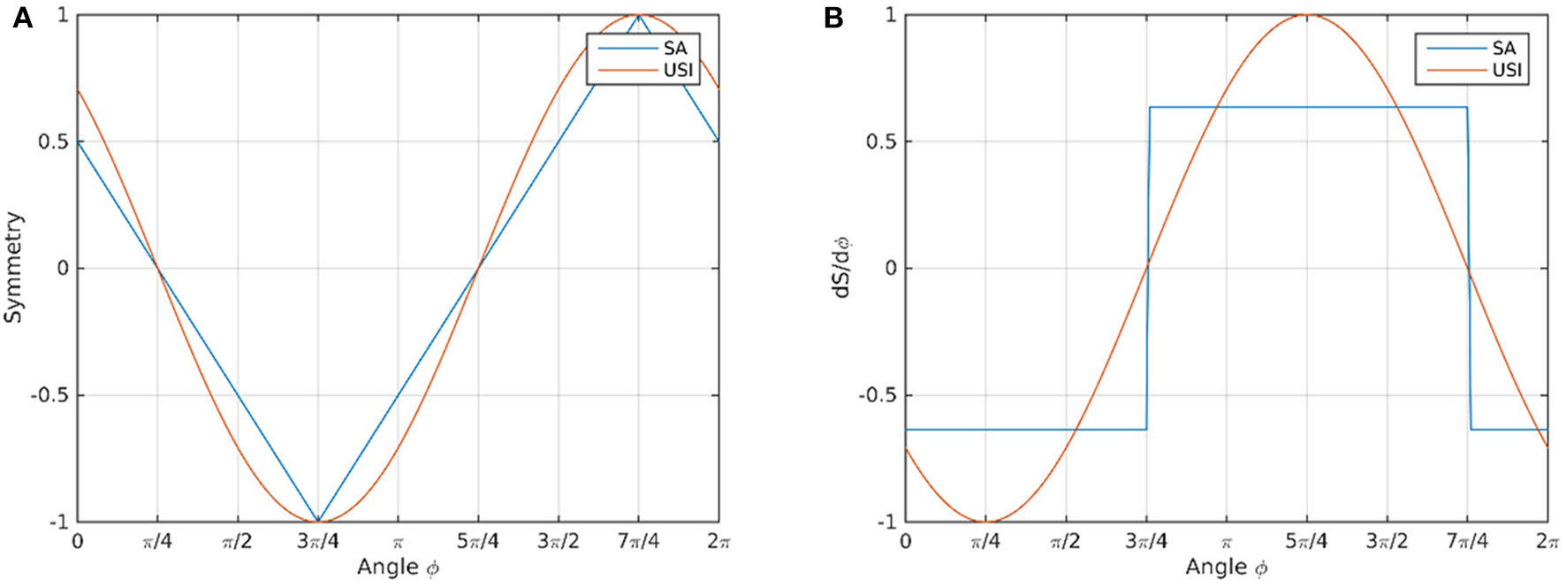
Methods *SA* (blue line) and *USI* methods (orange line) plotted as function of ϕ **(A)**; first derivatives (dS/dϕ) of the *SA* (blue line) and *USI* methods (orange line) **(B)**. The discontinuities can be observed in the derivatives panel for the *SA* method, occuring at ϕ = 3π/4 and ϕ = 7π/4.

To visualize the behavior of *SA* and *USI*, the methods were plotted as functions of φ as described in Equations (21, 22) in Figure 2A, with the respective first derivatives (dS/dϕ) in Figure 2B.

For signals that did not cross zero (for 0 < ϕ < π/2), the *SA* was found to be the best method: it returned a linear symmetry outcome, whereas the *USI* method slightly overestimated the symmetry outcome. On the other hand, for signals that crossed zero (for 0 < ϕ < 2π), the *SA* method exhibits discontinuities in the derivatives at ϕ = 3π/4 and ϕ = 7π/4. These discontinuities are an artificial property of the *SA* method and do not reflect a sudden change of a signal moving continuously from positive to negative or *vice versa*. The *USI* method did not yield any discontinuities but its behavior was not linear and slightly overestimated the symmetry measure. The *USI* method may be interpreted as a continuous approximation to *SA*, since $USI = sin\left(\frac{\pi }{4}SA\;\right)$.

**Figure 3:**
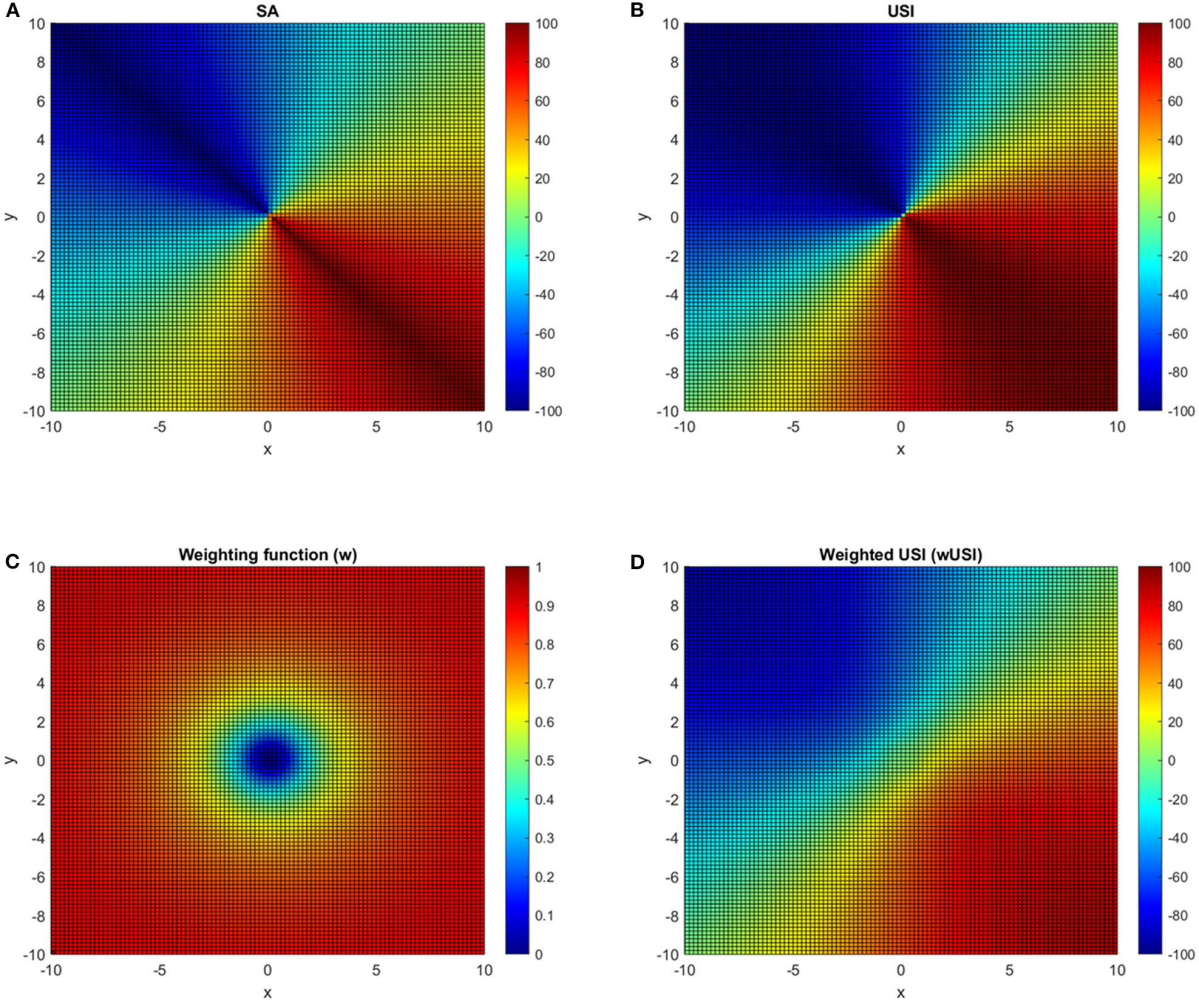
Heat maps for the *SA* **(A)**, *USI* **(B)** methods, for the weighting function based on a profile similar as a Cauchy distribution **(C)** and for the *wUSI* method with the weighting factor applied **(D)**. The three methods output **(A,B,D)** are on a common scale of [−100, 100] and the input signals *x, y* have a range of [−10, 10], being σ set to 1. A symmetry value of zero (green color) represents a perfect symmetry scenario and a symmetry value of 100 or −100 (dark red and dark blue color, respectively) represents a scenario of complete asymmetry. A detailed description of the weighting function application can be found in the Supplementary Material.

To avoid artificial inflation, the weighting function (Equation 24) was applied to the *USI* method, yielding the *wUSI* method. The behavior of *SA, USI*, the weighting function *W*(*x, y*, σ) and of *wUSI* are depicted in the heat maps, in Figures 3A–D, respectively. Both *SA* and *USI* methods (Figures 3A,B), which are applicable to positive and negative values, exhibit a scaling invariance due to axiom (Equation 5), i.e., they depend only on the ratio of *x* and *y*. With the application of the weighting factor, *W* (Figure 3C) this property is weakened in the near of the center, as seen in Figure 3D. In this region, both *x* and *y* are small and may be more affected by errors. The weighting application reduces this, as seen in Figure 3D.

### Application of Symmetry Methods to Physiological Data

For both discrete and continuous applications of the *wUSI* method, a 0.5% BW value was defined for the σ value in the weighting function. This value was based on the most sensitive component of the three-dimensional GRF (Fx), from which the minimum standard deviation was calculated. Thus, artificial inflation could be avoided, avoiding filtering of real features of the symmetry results. More details may be found in the Supplementary Material.

**Figure 4:**
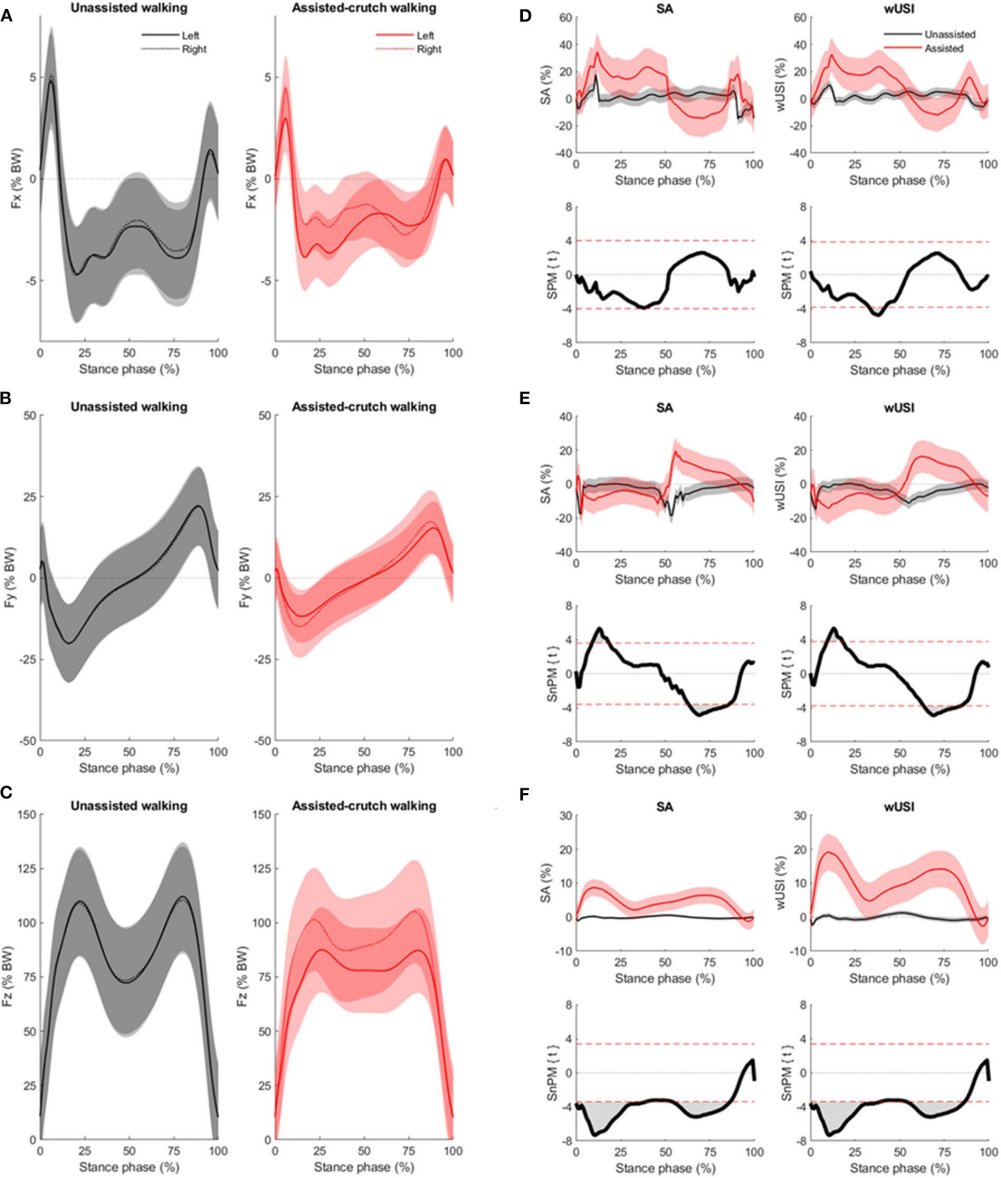
Mean curves (bold) with standard deviation (shaded) for the Fx **(A)**, Fy **(B)** and Fz **(C)** components of the GRF for the unassisted walking (black) and crutch-assisted (red) walking condition for the left (solid line) and right (dotted line) side. Mean curves (bold) with standard deviation (shaded) of asymmetry results using the *SA* and *wUSI* methods with the continuous approach application for the unassisted (black line) and crutch-assisted (red line) walking condition are presented in the top row of panels **(D–F)**, for the Fx, Fy, and Fz components of the GRF, respectively. Positive values of both *SA* and *wUSI* indicate higher GRF values yielded on the right side, whereas negative values indicate higher GRF values yielded on the left side, for each component. The bottom rows of panels **(D–F)**, represent the two-sample *t*-test statistical parametric or non-parametric mapping trajectories, SPM{t} and SnPM{t}, for the respective symmetry method and GRF component. Horizontal dashed red lines on the SPM panels indicate the critical thresholds (*z*-star values) for significance. As a two-tailed *t*-test was applied, positive and negative *z*-star values were yielded.

The mean curves and standard deviations of the Fx, Fy, and Fz GRF components during both unassisted and crutch-assisted walking conditions are depicted in Figures 4A–C, respectively. Each plot only included the stance phase (0–100%) of the gait cycle for both limbs (represented by continuous and dotted lines for left and right limb, respectively). During the crutch-assisted walking condition (plotted on the right-hand side of panels of Figures 4A–C for Fx, Fy, and Fz, respectively), all the participants used the crutch on their dominant hand (right hand) to where the participants' offloaded a maximum of 18.0 ± 6.5% BW (mean ± standard deviation).

The results of the asymmetry data calculation for the three components of the GRF using the *SA* and *wUSI* methods with the discrete approach, are found in Table 1. Parametric *t*-tests were applied as all discrete approach asymmetry results followed a normal distribution. Significant differences between walking conditions in the Fy and Fz component of the GRF for both methods were detected (Table 1, *p* < 0.001). However, no differences were found in the Fx component (Table 1, *p* = 0.073).

**Table 1:** Comparison of participants' (*n* = 15) asymmetry differences between walking conditions in the three components of the GRF, for both methods applied (*SA* and *wUSI*) when applying a discrete approach.

	***SA***				***wUSI***			
	**Unassisted**	**Crutch-assisted**	***p-*****value**^**a**^	**Cohen's d**	**Unassisted**	**Crutch-assisted**	***p-*****value**^**a**^	**Cohen's d**
**Fx**	−1.1 ± 5.9	−5.4 ± 8.0	0.073	0.5	−1.7 ± 9.2	−8.4 ± 12.4	0.073	0.5
**Fy**	−0.3 ± 1.4	5.6 ± 4.3	<0.001	−1.4	−0.5 ± 2.2	8.7 ± 6.7	<0.001	−1.4
**Fz**	−0.1 ± 0.6	4.9 ± 3.5	<0.001	−1.4	−0.2 ± 1.4	10.8 ± 7.8	<0.001	−1.4

a Student's t-test for homogenous variance, level of significance set at 0.05.

*Mean and standard deviation values for the SA and wUSI methods were computed using the impulse value for the right and left limb of each component. Positive values of both SA and wUSI indicate more offloading of the GRF component on the assisted left limb*.

For the continuous approach, the asymmetry data calculated by the *SA* and *wUSI* methods for the Fx component (Figure 4D) of the GRF followed a normal distribution. The asymmetry data for the Fy component (Figure 4E) only followed a normal distribution when calculated with the *wUSI* method. Asymmetry data for the Fz component (Figure 4F) did not follow a normal distribution with both methods. For all GRF components, the absolute *z*-star value, the significant clusters' start and end points, and the respective *p*-value for the *SA* and *wUSI* methods are depicted in Table 2.

**Table 2:** Values for *z*-star, clusters of significance (regarding % of stance phase) and *p*-values for the SPM comparison results when applying the *SA* and *wUSI* methods for the three components of the GRF when applying a continuous approach.

**Method**	**Component**	***z*-star**	**Clusters of significance (% stance phase)**	***p*-value**
*SA*	Fx	4.0	n/a	*p* > 0.05
	Fy	3.6	8.9–18.5	*p* = 0.002
			63.0–85.7	*p* <0.001
	Fz	3.4	0.0–39.3	*p* < 0.001
			53.8–86.4	*p* < 0.001
*wUSI*	Fx	3.8	32.9–41.9	*p* < 0.001
	Fy	3.8	9.3–18.0	*p* = 0.002
			63.4–84.4	*p* < 0.001
	Fz	3.4	0.0–39.3	*p* < 0.001
			53.8–86.4	*p* < 0.001

Parametric paired *t*-tests performed with SPM yielded a single significant cluster in Fx for the *wUSI* and no significant clusters were found for *SA* (Figure 4D). Two significant clusters were found in Fy (Figure 4E) following a parametric *t*-test for *wUSI* and a non-parametric *t*-test for *SA*. Similarly, non-parametric *t*-tests performed for both *wUSI* and *SA* yielded two significant clusters each (Figure 4F). Identified significant clusters following SPM comparison or *SA* and *wUSI* (Figures 4D–F) are summarized and listed in Table 2. For all significant clusters, higher significant asymmetry values for the crutch-assisted walking condition was detected.

## Discussion

The *wUSI*, a hereby newly proposed method, was capable to identify gait asymmetries across all three GRF components. Furthermore, the five axioms presented here were used as a benchmark for determining adequate gait asymmetry methods and addressed inconsistencies that exist in the literature.

The hypothesis of this study was supported with use of the *wUSI* method, as it was able to identify higher asymmetries in all three GRF components during crutch-assisted walking. Though considered an appropriate method, use of the *SA* method partially supported our hypothesis, as higher asymmetries were identified in only two of three GRF components during crutch-assisted walking. A reliable identification of gait asymmetry across multiple planes is essential in clinical settings, as gait asymmetry in musculoskeletal conditions typically leads to complex, multiplanar changes in forward movement.

The identification of asymmetries in the Fx, Fy, and Fz components of the GRF as shown here (Figures 4D–F, respectively) may be useful to understand multiplanar gait adaptations following treatment or intervention. Particularly in the Fx component of the GRF, reductions in this component found during mid-stance in crutch-assisted walking are likely influenced by reduced velocity and decreased magnitude to maintain balance (Li et al., 2001). In initial mid-stance, a transit from double- to single-limb support occurs. Mediolateral alterations during this time of stance have been identified as a strategy used to maintain dynamic balance (Raja et al., 2012) and avoid falling (Åberg et al., 2010). The ability to identify asymmetry in this component as shown here (Figure 4D) may thus be able to provide additional information on these strategies for fall prevention (Hendrickson et al., 2014; Beyaert et al., 2015). Only when applying the *wUSI* with the continuous approach to this component, higher asymmetries in crutch-assisted walking were found compared to the unassisted walking condition (Figure 4D). This was not identified using the *SA* method in application of both continuous and discrete approaches nor using the *wUSI* in application of the discrete approach. Furthermore, discrete approaches may also introduce bias due to the variable selection (e.g., maximum values that may have different temporal aspect at each step), which should be accounted for in further investigations. Thus, utilization of the *wUSI* with the continuous approach in clinical gait analysis to investigate asymmetries in the Fx component may provide additional information on frontal plane stability and on compensation strategies for fall prevention (Hendrickson et al., 2014; Beyaert et al., 2015).

Both *SA* and *wUSI* methods could detect significant asymmetry differences between walking conditions in defined regions of the Fy and Fz components. Regardless of the approach selected, both methods yielded significantly higher asymmetries for the crutch-assisted walking condition (Figures 4E,F, respectively). In the Fy component of the GRF for the crutch-assisted walking condition, the *SA* and *wUSI* found that the supported left limb exhibited both lower braking (at ~25% of the stance phase) and propulsion forces (at ~75% of the stance phase) compared to the unsupported right limb (Figures 4B,E). The supported left limb also exhibited lower vertical forces during the majority of the stance phase, excluding midstance (Figures 4C,F). These results support previous findings by Li et al. who also reported reductions in Fy during the braking and propulsion phases, as well as reduced magnitudes and a flattened mid-stance phase in Fz on the supported limb during crutch-assisted gait in healthy persons (Li et al., 2001). External walking aids assist with forward propulsion, decreasing the braking and propulsive forces regardless of crutch technique. However, this previous study investigated a different crutch gait, and thus differences in the vertical GRF during assisted gait are not directly comparable. A 3-point gait offloads more to the crutch than a 2-point gait, as seen in a patient population (Damm et al., 2013). The application of both the *SA* and the *wUSI* using the continuous approach allowed for the identification of asymmetries during specific stance phase regions, which could assess and compare the effects of rehabilitative treatment.

The commonly used *RI* and *SI* (Patterson et al., 2010; Błazkiewicz et al., 2014; Viteckova et al., 2018) did not fulfill the proposed symmetry axioms and were not considered as acceptable methods within this work. The utility of these methods has been debated: some state these methods do not present a clear advantage to discriminate abilities (Queen et al., 2020), others conclude that the method selection depends crucially on the variables of investigation (Patterson et al., 2010; Błazkiewicz et al., 2014). Within this work, both the *RI* and *SI* could not be used for asymmetry analyses, as they require signals that do not cross the zero axis. Only the *SA* and *wUSI* methods were found to satisfy all axioms and were independent of the input variable type.

Irrespective of the variable or signal type selected, a method must be mathematically robust to reliably provide asymmetry information. This may be achieved through the method's compliance with the axioms proposed, which were based on important biomechanical aspects that must be accounted for when investigating gait asymmetry. Until now, such criteria were neither formally established nor investigated. Recent work by Queen et al. described the importance of defining such mathematical aspects when determining a method for assessing gait asymmetry. Their proposed Normalized Symmetry Index was defined to return values across a defined range of data and that values of 0 and 100 (or −100) for scenarios of perfect symmetry and asymmetry, respectively (Queen et al., 2020). However, other aspects such as scaling invariance were not addressed. Other work has proposed new analysis methods and benchmarked them to available methods such as the *SI* (Zifchock et al., 2008), but a mathematical background that supports the method definition and robustness is lacking. By adhering to the axioms defined here, any method may be deemed as an appropriate method to determine gait asymmetry.

An appropriate method, i.e., compliant with the symmetry axioms, should be able to distinguish between two signals with known differences in asymmetry. For this purpose, repeated measurements of unassisted and crutch-assisted walking condition in healthy subjects were selected as input signals, for the symmetric and asymmetric condition, respectively. By using a third point of support with an elbow crutch, reductions in the three components of the GRF would happen, as previously reported by Li et al. (2001). To validate their methods, other studies have relied on models with variables from healthy and/or injured populations (Herzog et al., 1989; Winiarski et al., 2019; Queen et al., 2020), working with the assumption that healthy subjects exhibit symmetric patterns. Though asymmetry is commonly associated with injured populations (Patterson et al., 2010; Queen et al., 2014), healthy individuals also exhibit gait asymmetries (Sadeghi et al., 2000), which are accounted for within this investigation. Our results support this, indicating that when walking unassisted, healthy participants also exhibit some very minor GRF asymmetry (Figures 4D–F) in the three planes of motion. Thus, by mechanically ensuring the presence of symmetry and asymmetry in the different walking conditions across the three planes of motion, and performed by the same group of subjects, biases introduced by selecting population(s) to represent symmetric and asymmetric walking conditions, could be avoided.

Apart from the compliance of the symmetry methods to the axioms, a major contribution of the proposed method *wUSI* is the weighting function application in the continuous approach. With its application, artificial inflations on an individual level were avoided, without the consideration of very small differences in signal magnitude that near or cross the zero axis, particularly for the Fx component of the GRF. The artificial inflation found on an individual level can either mask asymmetries that may exist or yield asymmetries that do not exist on a group level. This is due to the dominance of such artificial inflations when summarized across the group. The developed weighting function avoids the creation of artificial inflation on the individual level, by applying targeted smoothing to the affected regions. Thus, on a group level, only specific asymmetry regions would be yielded, allowing for only true significant asymmetries in all GRF components to be identified. This is a critical advantage, since specific regions of the stance phase in each component correlate to different biomechanical functions (Richards et al., 2013; Webster and Darter, 2019).

A limitation of this study is the small asymmetry difference found between walking conditions in the Fx component. Since this investigation was performed with healthy individuals unaccustomed to crutch-assisted walking, deviations in the mediolateral component are low in magnitude and likely represent learning-relevant adaptations. Future studies should test the *wUSI* in highly variable GRF, such as acute rehabilitation settings, to investigate if pathological differences can also be identified. Another limitation of this study is that the signals of investigation were limited to GRF and did not include other signal types commonly collected during gait, such as kinematics. Though all three GRF components were considered, the *wUSI* remains to be tested for its utility for testing other parameters. Later work should address the applicability of *wUSI* to investigate asymmetry in kinematic or kinetic parameters.

The available symmetry methods discussed within this work were limited to those assessments that utilize of single variables as input. While other authors have proposed indices that may capture more comprehensive movement asymmetry (Hoerzer et al., 2015; Cabral et al., 2016), they require multiple movement variables to maximize their utility, such as kinematic and/or kinetic variables. Newly developed portable technologies allow for the collection of GRF forces to take place within clinical settings without the necessity of a large laboratory. As a result, GRF asymmetry can be determined at a very early time point following intervention or injury. Application of the *wUSI* with a continuous approach within these settings could assist in identifying specific asymmetric phases of weight bearing during rehabilitation. Furthermore, the ability of the *wUSI* to avoid artificial inflation is advantageous when analyzing other signals that cross zero, such as kinematics. The future development and establishment of a normative database for asymmetry deviations using the *wUSI* would allow for a benchmark against which patient populations could be compared. This would ultimately allow for increased utility of the *wUSI* within a clinical rehabilitation setting.

## Conclusion

In summary, the herein established symmetry axioms allowed for the definition of a robust and efficient symmetry method and enabled an evaluation against existing methods and a newly proposed weighted Universal Symmetry Index, *wUSI*, method. This analysis indicates that the *wUSI* method was suitable for the analysis of GRF asymmetries. It reduced non-linearities and artificial inflation found in the previous methods and allowed for the analysis of data that crosses the zero axis. Finally, specific time windows during the stance phase of gait were identified during which higher asymmetry behaviors were identified for the three planes of motion. When dynamic activities such as gait are considered for asymmetry analysis, continuous approaches should be favored to account for dynamic adaptations over time that are often missed in discrete approaches. The *wUSI* can also be universally applied across different types of signals for investigation of asymmetry during dynamic activities. Due to its sensitivity, the *wUSI* could be considered as a sophisticated method to quantify pathological gait asymmetries, and potentially used as a tool to evaluate and compare functional recovery during rehabilitation.

## Data Availability Statement

The datasets generated for this study are available on request to the corresponding author.

## Ethics Statement

The studies involving human participants were reviewed and approved by Ethikkomission der Charité (EA1/079/17). The patients/participants provided their written informed consent to participate in this study. Written informed consent was obtained from the individual(s) for the publication of any potentially identifiable images or data included in this article.

## Author Contributions

SAA, RME, PCR, GND, and ANA contributed to the study conception and design. SAA collected and processed the physiological data. AB contributed to the physiological data processing. RME performed mathematical analyses of the symmetry methods. SAA drafted the manuscript with support from RME, GND, and ANA. All authors contributed to data analysis and interpretation of results before approving the final manuscript prior to submission.

## Conflict of Interest

The authors declare that the research was conducted in the absence of any commercial or financial relationships that could be construed as a potential conflict of interest.
