# Supplementary material for: Quantifying Asymmetry in Gait: The Weighted Universal Symmetry Index to Evaluate 3D Ground Reaction Forces
Source: Front Bioeng Biotechnol. 2020 Oct 23;8:579511. doi: 10.3389/fbioe.2020.579511 (PMC7644861; doi:10.3389/fbioe.2020.579511)
Supplement: Supplementary file 1 [file Data_Sheet_1.docx]

Supplementary Material

# Development of a weighted symmetry measure

Since all symmetry measures depend on the ratio between two signals, $x$ and $y$ (e.g. $y/x$), its computation may exhibit irregular behavior if one or both signals are very small in magnitude compared to the precision of the measurement device. This produces what is here defined as “artificial inflation”. Under such circumstances, the ratio $y/x$ may have a very large value or values with changing signs, which do not correspond to real symmetry values. To overcome this issue, we present herein a weighting function, which efficiently filters out any artificial values in the symmetry measure yielded by small $x$ or $y$ values.

To analyze the error propagation, i.e. the effect of the uncertainty in the variables $x$ and $y$ on the uncertainty in the symmetry function $S$, we treat both $x$ and $y$ as independent, normally distributed random variables with different means $X$ and $Y$ but with the same standard deviation $\sigma$.

For $USI$ and also other symmetry measures, it appears difficult to develop analytical expressions for the standard deviation of $S$. For $USI=\frac{x-y}{\sqrt{2\left( x^{2}+y^{2} \right)}} ,$the distribution function of the numerator $x-y$ is a normally distributed random variable, with a mean $X-Y$ and a standard deviation $\sqrt{2} \sigma$. For the denominator, a non-central Chi distribution is obtained (Krishnamoorthy, 2016) with a mean and standard deviation that can be expressed only in terms of generalized Laguerre polynomials. For the ratio distribution, no closed or even any approximate expression exists. Thus, by sampling for a grid of values for $x$ and $y$, the standard deviation of the symmetry function is then computed as a function of $x$ and $y$ for $USI$ (Supplementary Figure 1). As expected, the highest accuracy for a fixed $x$ is obtained if $y=-x$, i.e., where $USI$ attains the minimum or maximum value. The function has its maximum of $1/\sqrt{2}$ at $x=y=0$, since for an arbitrary standard deviation $\sigma\neq0$, *USI* converges to this value, if $x,y$ converge to zero. This can easily be proven, for example, by a series expansion.

The shape reconstruction of the function in Supplementary Figure 1A was then attempted by means of an analytical expression, ($C$). A nearly optimal similarity between the computed numerical representation of the standard deviation and the analytic expression was found with the equation (1):

$WC\left( x,y,\sigma\right)=\frac{\sigma}{\sqrt{2\sigma^{2}+x^{2}+y^{2}}}$ (1)

Equation (1) resembles to a two-dimensional Cauchy distribution (Krishnamoorthy, 2016). Cauchy distributions are examples for heavy-tailed distributions, whose tails are not exponentially bounded. Note that $C$ can also be written as a function of the ratio $\sigma/x$ and $\sigma/y$. Thus, $C$ is only dependent on the ratio of the standard deviation $\sigma$ and the variables $x$ and $y$.


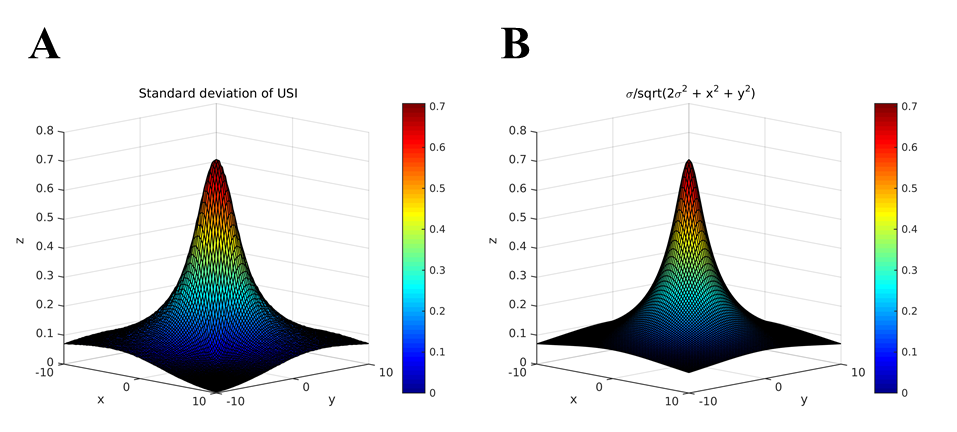


**Supplementary Figure 1 –** Numerical results for the functional relationship of the standard deviation of $USI$ as a function of $x, y$ by sampling normal distributed values at each grid point in the$x,y$-plane, $x, y\in\left[ -10,10 \right], \sigma=1$, sampling rate 10000 (A). Reconstructed profile of the relationship by the function $C$, equation (1) (B). For visualization purposes, the standard deviation $\sigma$ was set to a relatively large value (10% of the $x, y$ maximum values).

With the weighting function $W\left( x,y,\sigma\right)=1-\sqrt{2} C\left( x,y,\sigma\right)$, irregular peaks due to measurement errors can be filtered. Given an estimate $\sigma$ for the standard deviation of the experimental values $x$ and $y$, the weighted *USI* (*wUSI*) is defined in equation (2).

$wUSI\left( x,y,\sigma\right) = USI\left( x,y \right) *W\left( x,y,\sigma\right)= USI\left( x,y \right)*\left( 1 -\frac{\sqrt{2} \sigma}{\sqrt{2\sigma^{2}+x^{2}+y^{2}}} \right)$ (2)

# Choice of sigma value ($\boldsymbol{\sigma}$)based in the physiological signals

The value for the variable $\sigma$ should be represented by a smaller value than the standard deviation of the experimental data. Larger values may lead to a smoothing of characteristic properties of the computed symmetry function, becoming particularly considerable if $\sqrt{x^{2}+y^{2}}<5\sigma$. Thus, the definition of the $\sigma$ value enables a flexible calibration of the *wUSI*.

To estimate a $\sigma$ value for the current dataset, the most sensitive component of the ground reaction forces (GRF), Fx, was selected. To achieve it, the mean standard deviation for this component data for both walking conditions was computed (Supplementary Figure 2) and the minimum value for each signal extracted. For the unassisted walking condition the values are 0.4549 and 0.4873 %BW for left and right side, respectively and for the crutch-assisted walking condition the values are 0.4920 and 0.4248 %BW for left and right side, respectively. Based on these values, 0.5 %BW was defined as $\sigma$ value for the current dataset and applied in Equation 2.

The performance and efficiency of the weighting function is demonstrated by the examples in Section 3 of this Supplementary Material.


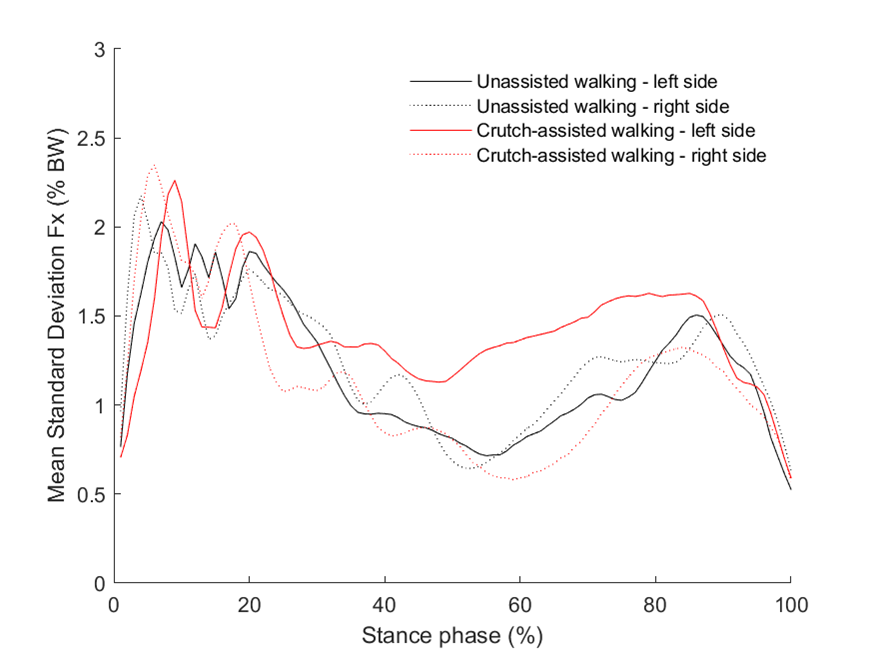


**Supplementary Figure 2 –** Mean Standard Deviation (% Body Weight, BW) of the physiological signals for the Fx components of the GRF for the unassisted (black) and crutch-assisted (red) walking conditions, for the left (solid line) and right (dotted line) side. Based on the minimum values for the mean standard deviation of each signal, a value of 0.5 %BW was defined as $\sigma$ value for the current dataset to be applied in Equation 2.

# Individual participant example

A sample of individual participant’s (participant 01) mean curves of the physiological signals for the three components (Fx – mediolateral, Fy – anteroposterior and Fz – vertical) of the GRF were selected to demonstrate the performance and efficiency of the *wUSI* method (Supplementary Figure 3).


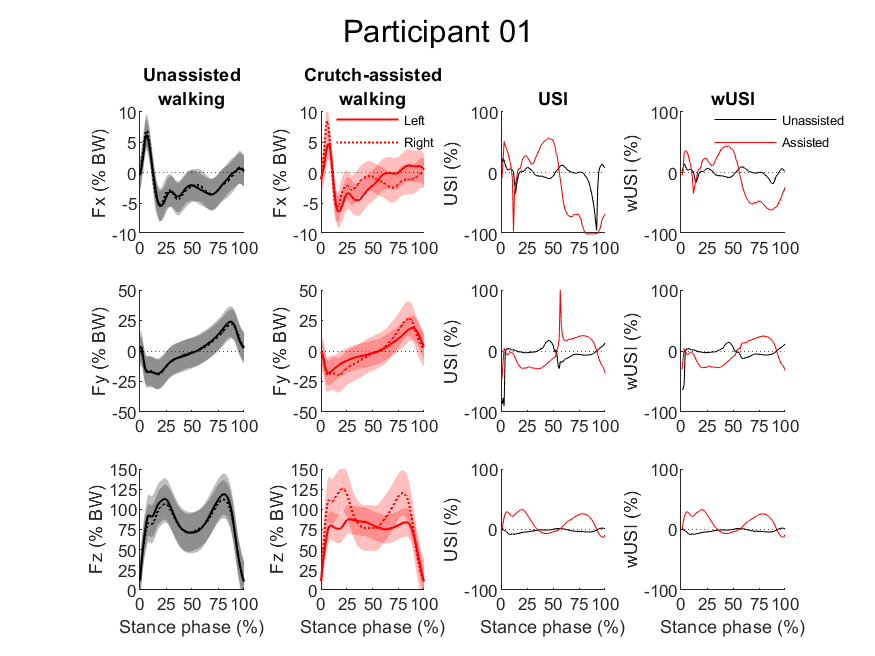


**Supplementary Figure 3 –** Sample of individual participant’s (participant 01) mean curves (bold) with standard deviation (shaded) of physiological signals for the Fx (first panel row), Fy (second panel row) and Fz (third panel row) components of the GRF for the unassisted (black) and crutch-assisted (red) walking condition for the left (solid line) and right (dotted line) side. Mean curves of asymmetry results using the *USI* and *wUSI* methods for the unassisted (black line) and crutch-assisted (red line) walking condition are represented in the third and fourth column panels, respectively. Positive *USI* and *wUSI* values indicate more offloading of the GRF component on the assisted limb.

# References

Krishnamoorthy, K. (2016). *Handbook of Statistical Distributions with Applications*. 2nd ed. New York: Chapman and Hall/CRC doi:10.1201/b19191.
